# Brisk Erythematous Reaction Outside of the Radiation Field in a Patient Treated With Adjuvant Radiation for Phyllodes Tumor

**DOI:** 10.1016/j.adro.2021.100752

**Published:** 2021-07-14

**Authors:** Aarushi Kalra, Lindsay Dehr, Timothy D. Malouff, Byron C. May, Laura A. Vallow

**Affiliations:** a Philadelphia College of Osteopathic Medicine-Georgia Campus, Suwannee, Georgia; b Department of Radiation Oncology, Mayo Clinic, Jacksonville, Florida

## Introduction

Phyllodes tumor of the breast is a rare connective tissue disorder. Although usually benign, these tumors have the potential to metastasize.^1^ The role of adjuvant radiation therapy is controversial, as studies have been inconclusive if there is an overall survival benefit to adjuvant treatment.^2^^,^^3^

Furthermore, systemic lupus erythematosus (SLE) is a relatively common collagen vascular disease that is considered by some to be a relative contraindication to radiation therapy given concerns of normal tissue toxicities, although recent studies have suggested that radiation therapy can be given safely.^4^^,^^5^ Our case report discusses a patient treated with phyllodes tumor of the breast with a brisk erythematous reaction, similar to radiation dermatitis, but occurring outside of the treatment field and to a more severe degree than expected.

## Case Presentation

Our patient is a 60-year-old Caucasian woman with a medical history significant for SLE without organ involvement and a benign left breast mass status postexcision in 2006. She was in her usual state of health until screening diagnostic mammogram demonstrated an increased conspicuity of a bilobed nodule within the upper outer quadrant of the left breast. Ultrasound of the left breast demonstrated a 2.3 × 0.8 × 1.9 cm mixed echogenicity partially cystic and partially solid appearing mass 2:00, 9 cm from nipple correlating with bilobed mammographic finding. Given the benign appearance of the mass, short-term follow-up was recommended. The mass persisted on follow-up, and a biopsy was recommended. Pathology from the biopsy revealed a fibroepithelial lesion with features suggestive of phyllodes tumor. She was referred to general surgery and complete surgical excision was recommended. She underwent partial mastectomy, with final pathology demonstrating a 2.5 cm low-grade malignant phyllodes tumor with positive margins. She was taken back to the operating room several days later for re-excision of margins, with pathology indicating no residual tumor. At the time of surgery, the SLE was reasonably well controlled, although she had chronic lesions involving the scalp, torso, and upper extremities, all out of the future radiation treatment field, with limited response to systemic therapy.

The patient tolerated both operations well and presented to the radiation oncology department for discussion of adjuvant therapy. The role of adjuvant radiation therapy was discussed at length, and it was explained to her that given the rarity of the tumor, the role of adjuvant radiation therapy is not well defined.^6^ We discussed the potential local control benefit with adjuvant radiation therapy, although this was uncertain, and studies have suggested no overall survival benefit with adjuvant treatment.^7^ If she were to develop a local recurrence after resection alone, salvage treatment options of surgery and radiation therapy were discussed. Considering her history of chronic cutaneous lupus, she was advised that there was a higher risk of acute and chronic skin toxicity from radiation therapy. After a full discussion, observation was recommended given the SLE as well as the reasonable salvage options available, although she elected to proceed with adjuvant radiation therapy to reduce the risk for local recurrence.

**Figure 1:**
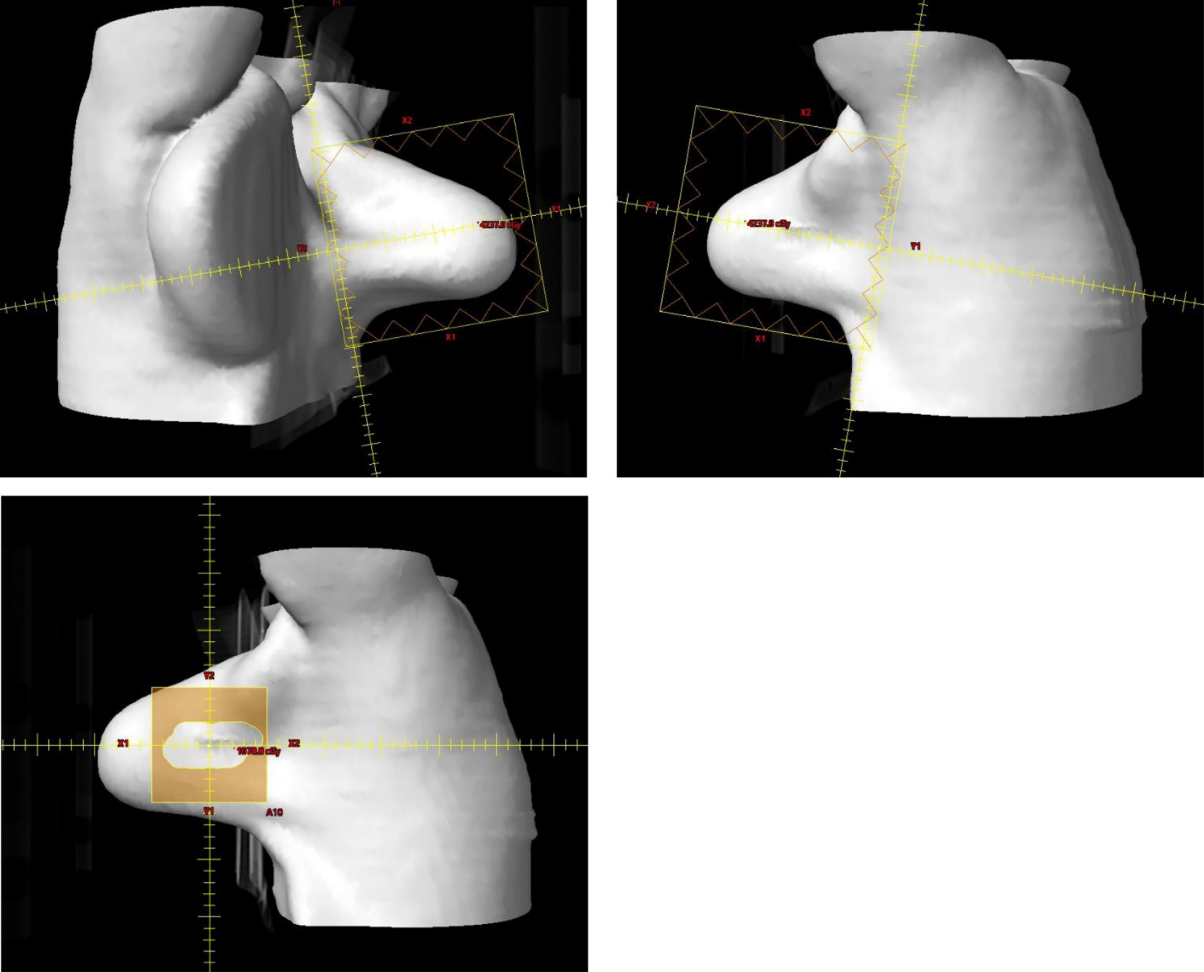
Beams-eye view of the (A) medial tangent, (B) lateral tangent, and (C) boost volume.

The patient was treated in the prone position to a dose of 40 Gy in 15 fractions to the whole breast followed by a 10 Gy boost in 5 fractions. This fractionation and rationale for boost were based on our institutional standards for treatment of breast cancer. For the whole breast treatment, 2 standard oblique tangents and 6 MV photons were used. A field-in-field technique was used to improve dose homogeneity, with a hotspot of approximately 106%. Given the cavity was on the lateral right breast, a 10 Gy boost using a single en-face 16 MeV electron beam without bolus was used (Fig 1).

**Figure 2:**
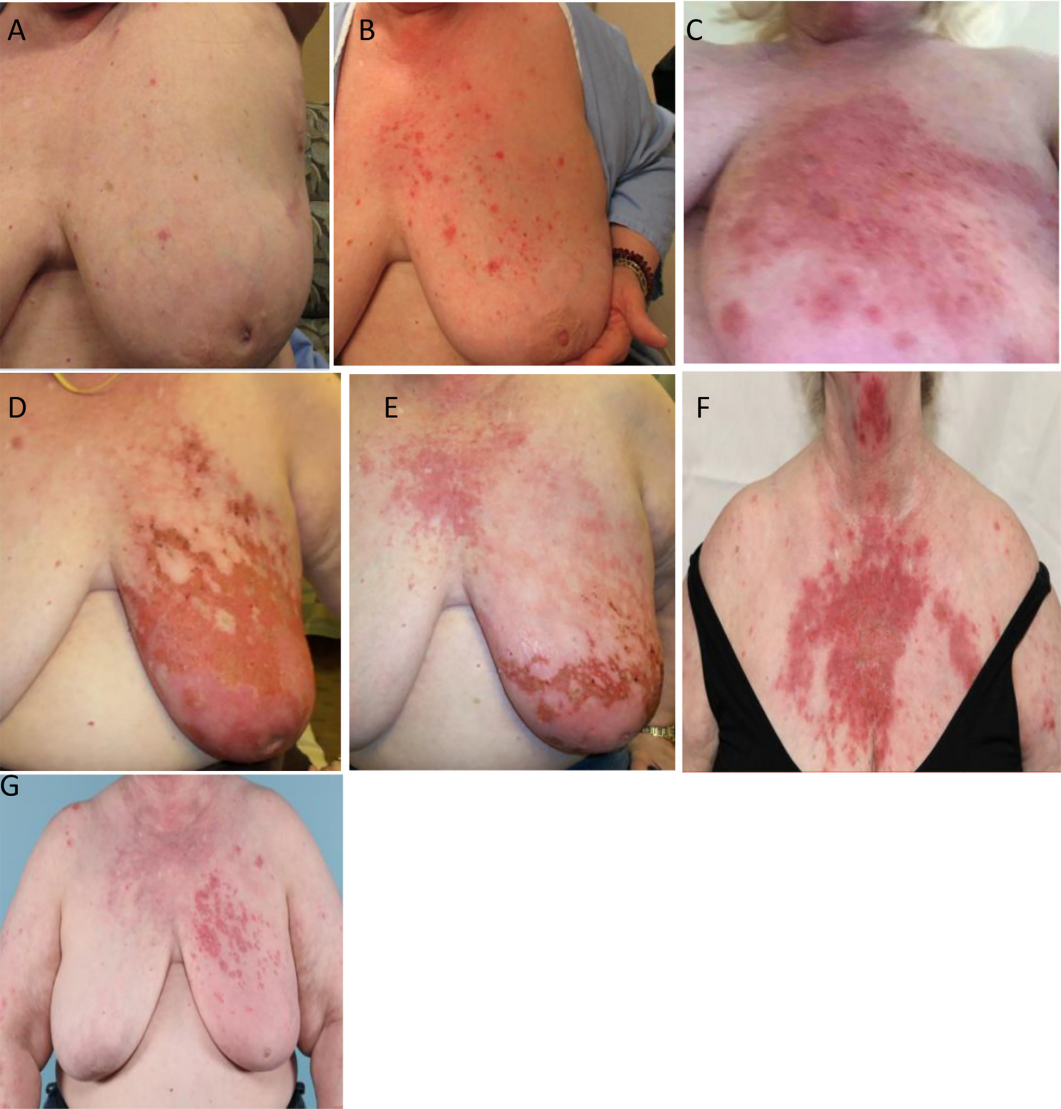
Radiation dermatitis at various time points. (A) Fraction 4/15, (B) fraction 10/15, (C) 1 week after completion, (D) 3 weeks after completion, (E) 1 month after completion, (F) 5 weeks after completion, and (G) 3.5 months after the completion of radiation.

She was treated concurrently with 200 mg of hydroxychloroquine 2 times daily for treatment of SLE during radiation, as directed by dermatology. The patient's skin was closely monitored throughout her treatment course by a multidisciplinary team of dermatology and radiation oncology and photos were taken periodically. The course of treatment was tolerated moderately well; she experienced acute toxicities of pain, edema, brisk erythema, blistering, and dry and moist desquamation, consistent with expected grade 3 radiation dermatitis within the treatment field. Unexpectedly, she developed a diffuse erythematous, painful rash across the anterior chest wall extending up to the central neck and chin with scattered bullae, which was outside of the treated site (Fig 2). This required opioid analgesic for pain control, oral antibiotic for suspicion of skin infection, a slow steroid taper beginning at 60 mg daily, frequent Domeboro soaks, and application of topical agents such as Miaderm and silver sulfadiazine cream.

At 4 months after completion of treatment, the acute radiation dermatitis reaction within the treatment field had resolved; however, scattered erythematous plaques and scaling on the bilateral breast, anterior chest wall, and bilateral upper extremities persisted. After multidisciplinary discussion, this was felt to be secondary to lupus flare aggravated by radiation, although the etiology was uncertain. At last follow-up, she continued to recover well and was without mammographic findings concerning for malignancy.

## Discussion

This patient presented with a phyllodes tumor, a rare connective tissue tumor located in the breasts, which has the potential to metastasize despite being most frequently benign. The first line of treatment for malignant phyllodesis is lumpectomy without adjuvant local therapies. Adjuvant radiation therapy is typically not recommended owing to the lack of long-term effectiveness shown in multiple studies; however, this therapy is usually administered depending on the individual's situation. To date, the local control benefit of adjuvant radiation therapy is unclear. A study by Belkacémi et al^2^ found that adjuvant radiation therapy improves the local control for borderline and malignant tumors from 59% to 86% at 10 years, although no local control benefit was found in the study by Mitús et al^3^ comparing observation or adjuvant radiation based on margin status. Recently, a meta-analysis of 17 studies showed improvement in the local recurrence rate with adjuvant radiation therapy, as well as low rates of metastatic disease.^8^

As expected, the patient experienced radiation dermatitis within the treatment field. Notably, she developed a brisk erythematous reaction outside of the treatment field concurrently with the expected radiation dermatitis, with the findings felt to be consistent with a lupus reaction. One hypothesis regadring the severity is symptoms is related to the interplay of estrogen in SLE. To support this hypothesis, studies have suggested that estrogen has large effects on cytokine expression along with estrogen receptor change enhancements in patients with lupus.^9^ The interaction of estrogen and SLE could have increased the cutaneous symptoms of SLE throughout the resection and radiation period, leading to a higher probability of the spread of dermatitis. Along with estrogen, obesity and fat deposition in the breasts are linked to lupus symptom exacerbations leading to a greater probability of the cutaneous spread of the rash. However, these conditions can be considered as mild contraindications for radiation and could be one of the main damaging components behind the acute high-grade dermatitis outside of the radiation field.

SLE is considered a risk factor for radiation-induced adverse effects and is considered a relative contraindication to radiation therapy, and the delivery of radiation therapy in patients with SLE remains controversial. Chen et al^4^ reported on 36 patients with collagen vascular diseases (including 5 with SLE) treated with breast conserving surgery. Although there were increased late toxicities in the collagen vascular disease group, there were no differences in acute toxicities. One case study was done on a female SLE patient with breast cancer where she received radiation therapy and developed grade 3 dermatitis, but did not experience any major side effects that were vastly different from a patient with no autoimmune conditions.^10^ Another study at the Toronto Lupus Clinic followed patients for 29 years who received radiation therapy despite their underlying conditions and concluded that no one developed any statistically significant acute or long-term toxicity.^11^ These are a few examples, but because there are limited data on the relationship between patients with SLE and radiation dermatitis, it is still advised to use radiation with caution in an individual case-by-case scenario. For instance, a study done on radiation therapy effects in collagen vascular disease showed a higher risk of acute toxicity in the breasts compared with lower extremity radiation,^12^ and radiation reaction in SLE may be similarly site dependent. Based on these findings and other sources, there are no absolute restrictions for radiation therapy in patients with SLE, but a higher possibility of developing adverse reactions earlier. Therefore, the low dosage treatment was requested by the patient after considering other alternatives.^5^

For patients with collagen vascular diseases, partial breast irradiation (PBI) is a potential option to minimize the volume of normal tissue receiving low-dose irradiation by limiting the target volume to a small margin, as opposed to whole breast irradiation. There are multiple approaches to PBI, including external beam, interstitial implants, and balloon-based brachytherapy, with the efficacy and safety of PBI demonstrated in several trials.^13^^,^^14^ Although PBI has not been studied extensively in patients with connective tissue diseases, there is a theoretical advantage to reducing toxicities by reducing the integral dose. This approach was not performed on this patient given our institutional standard to treat the entire breast in most patients. Further studies are needed to determine whether PBI can improve toxicities in this population.

The exact mechanism of the radiation-induced lupus flare remains uncertain. One hypothesis is that the autoimmune reaction of SLE was possibly triggered locally due to the activation of an autoantigen recognizing potential inflammation after radiation was administered the first time.^15^ Another possible explanation is a previously unreported reaction with hydroxychloroquine and radiation, similar to a radiation recall seen with antineoplastics.^16^ One case study also showed a patient with SLE who developed “pruritus and cutaneous reactions like rash, exanthematous pustulosis, and urticaria” after antimalarial administration. These adverse effects seem to appear around 10% to 20% of the time.^17^ Another possible explanation for her extensive rash is ultraviolet sensitivity, which is a well-described adverse effect of hydroxychloroquine.^17^ As the rash appeared primarily in a sun-exposed area, there may have been a synergistic effect of therapeutic radiation, which often increases the probability of sunburns, and hydroxychloroquine-induced ultraviolet sensitivity, causing the severe dermatitis. Usually patients with SLE present with chronic radiation dermatitis consequences; however, hydroxychloroquine could have accelerated the timing of the dermatitis’ appearance and spread of a hydroxychloroquine-induced erythema multiforme reaction.^18^

## Conclusions

Although radiation therapy in patients with SLE is generally safe, this case demonstrates a robust skin reaction outside of the treatment field, likely secondary to a radiation-induced SLE flare or a rare interaction with hydroxychloroquine. Further studies on this phenomenon could provide more information and support on this issue of dermatitis outside the original field of radiation and the acute nature of the reaction.
